# Hyperintense signals in cerebral blood flow maps acquired with pseudo-continuous arterial spin labeling MRI in mice

**DOI:** 10.64898/2025.12.02.691929

**Published:** 2025-12-05

**Authors:** Xiuli Yang, Yuguo Li, Adnan Bibic, Zhiliang Wei

## Abstract

**Background and Purpose:**

Pseudo-continuous arterial spin labeling (pCASL) MRI is a widely used, noninvasive, contrast-agent-free technique for measuring cerebral blood flow (CBF) and assessing vascular dysfunction across diverse clinical settings and murine disease models. In practice, arterial-transit artifacts that generate hyperintense signal in CBF maps warrant careful consideration. While these effects are well characterized in humans, they are less well understood in mice owing to the marked interspecies physiological differences.

**Methods:**

To address this knowledge gap, we systematically characterized pCASL hyperintense signal as a function of post-labeling delay (PLD) and crusher-gradient strength in mice. Numerical simulations were also performed to validate the experimental findings.

**Results:**

We found that hyperintense signals in mice extend to arteries, major veins, and ventricular structures (e.g., choroid plexus). Such a pattern was different from human pCASL images, where hyperintense signals are predominantly present in arteries. Statistical analyses supported a PLD of 500 ms as a pragmatic balance between detection sensitivity and suppression of vascular contamination. Additional experiments and numerical simulations showed that, within the tested range, stronger crusher gradients provided little extra vascular suppression—primarily because large vessel calibers relative to small voxels limit intravoxel phase dispersion. These findings refine the interpretation of murine pCASL signals and facilitate more accurate perfusion imaging in preclinical pathophysiological studies.

## Full Text Availability

The license terms selected by the author(s) for this preprint version do not permit archiving in PMC. The full text is available from the preprint server.

